# Phosphoglycerate kinase is a central leverage point in Parkinson’s disease–driven neuronal metabolic deficits

**DOI:** 10.1126/sciadv.adn6016

**Published:** 2024-08-21

**Authors:** Alexandros C. Kokotos, Aldana M. Antoniazzi, Santiago R. Unda, Myung Soo Ko, Daehun Park, David Eliezer, Michael G. Kaplitt, Pietro De Camilli, Timothy A. Ryan

**Affiliations:** ^1^ Department of Biochemistry, Weill Cornell Medicine, New York, NY 10065, USA.; ^2^ Aligning Science Across Parkinson’s (ASAP) Collaborative Research Network, Chevy Chase, MD 20815, USA.; ^3^ Department of Neurosurgery, Weill Cornell Medicine, New York, NY 10065, USA.; ^4^ Departments of Neuroscience and Cell Biology, Howard Hughes Medical Institute, Program in Cellular Neuroscience, Neurodegeneration and Repair, Yale University School of Medicine, New Haven, CT 06520, USA.

## Abstract

Although certain drivers of familial Parkinson’s disease (PD) compromise mitochondrial integrity, whether metabolic deficits underly other idiopathic or genetic origins of PD is unclear. Here, we demonstrate that phosphoglycerate kinase 1 (PGK1), a gene in the PARK12 susceptibility locus, is rate limiting in neuronal glycolysis and that modestly increasing PGK1 expression boosts neuronal adenosine 5′-triphosphate production kinetics that is sufficient to suppress PARK20-driven synaptic dysfunction. We found that this activity enhancement depends on the molecular chaperone PARK7/DJ-1, whose loss of function significantly disrupts axonal bioenergetics. In vivo, viral expression of PGK1 confers protection of striatal dopamine axons against metabolic lesions. These data support the notion that bioenergetic deficits may underpin PD-associated pathologies and point to improving neuronal adenosine 5′-triphosphate production kinetics as a promising path forward in PD therapeutics.

## Teaser

PGK1 confers metabolic resilience to the brain.

## INTRODUCTION

The brain is a metabolically vulnerable organ suffering acute functional decline when fuel delivery is compromised. We previously showed that central nervous system nerve terminals rely on efficient activity-dependent up-regulation of adenosine 5′-triphosphate (ATP) synthesis to sustain function and undergo abrupt synaptic collapse when this process fails (*1*–*3*). Reduced fuel delivery to the brain is correlated with aging and is an early predictor of eventual neurological dysfunction (*4*), suggesting that as fuel delivery becomes compromised, synaptic function becomes increasingly vulnerable to genetic metabolic lesions. Parkinson’s disease (PD) has long-been thought to be, in part, driven by metabolic vulnerability of dopamine (DA) neurons of the substantia nigra pars compacta (SNc) as two of the earliest identified genetic drivers of PD, PARK2 (PARKIN) and PARK6 (PINK1), when mutated, compromise the integrity of mitochondria (*5*), a central hub of cellular bioenergetic support. Furthermore, SNc DA neurons, which fire constantly because of pacemaker activity, comprise extreme, elaborated axonal arbors, which, in turn, are thought to support up to 1 million DA release sites each in the human striatum, thus presenting a large metabolic burden (*6*). In addition, the striatum, where SNc axons project, is characterized by low abundance of glucose (*7*) and trophic factors. Many experimental animal models of PD specifically target bioenergetic pathways in DA neurons, as lesioning mitochondrial function in these neurons leads to their degeneration (*8*, *9*).

Several recent discoveries point to a critical but unexpected outsized role of the glycolytic enzyme phosphoglycerate kinase 1 (PGK1) in protecting neurons against neurological impairment. PGK1, the first ATP-producing enzyme in glycolysis, catalyzes the sixth step in this 10-step enzymatic cascade. A chemical screen of a subset of Food and Drug Administration–approved drugs capable of suppressing cell death identified terazosin (TZ) as a weak (~4%) off-target activator of PGK1 (*10*). TZ was subsequently shown to confer significant protection in numerous models of PD (mouse, rat, *Drosophila*, and human induced pluripotent stem cells) (*11*), implying that contrary to expectations, PGK1 activity is a critical modulator of glycolytic throughput. Clinical use of TZ for treatment of benign prostate hyperplasia provided data for a retrospective analysis, which showed that prolonged use of TZ reduced the risk of developing PD by up to ~37% compared to tamsulosin, whose chemical structure differs significantly from TZ but has the same molecular target (*12*). Although it was subsequently suggested that the impact of TZ versus tamsulosin on PD was due to a deleterious effect of the later drug (*13*), a more recent three-way analysis of different benign prostate hyperplasia treatments identified that TZ has beneficial effects (*14*). In addition, it is interesting to note that PGK1 is part of the PARK12 susceptibility locus (*15*) and certain PGK1 mutations in humans are characterized by early-onset PD (*16*–*18*).

These data all predict that PGK1 activity is a crucial leverage point in neuronal bioenergetic control and that bioenergetic deficits, in turn, underpin many forms of PD. Here, we demonstrate that PGK1 is the rate-limiting enzyme in axonal glycolysis and that modest changes in PGK1 activity accelerate neuronal ATP production kinetics capable of reversing the synaptic deficit driven by the PARK20/Synaptojanin 1 (Synj1) 1 mutation. We identified PARK7/DJ-1, the PD-associated molecular chaperone, as a necessary gene for PGK1 to up-regulate ATP production as loss of PARK7/DJ-1 itself led to deficits in neuronal glycolysis that impaired the ability of PGK1 up-regulation to provide protection. We showed that increasing PGK1 abundance in vivo offered strong protection against striatal DA axon dysfunction. These data strongly support the idea that PGK1 serves as a critical lever arm in controlling axonal bioenergetics and suggests that hypometabolic deficits may underlie a wide spectrum of PD etiology (*11*, *12*, *14*, *16*, *17*, *19*–*21*).

## RESULTS

### A genetic expression suppressor screen of hypometabolic synaptic failure identifies PGK1 as a rate-limiting enzyme in presynaptic bioenergetics

**Fig. 1.**
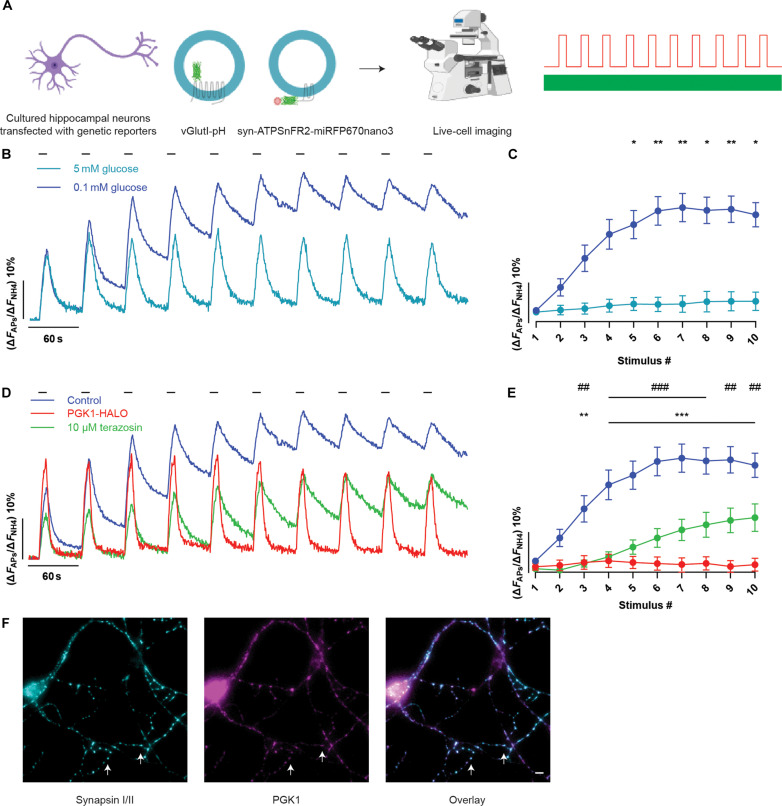
PGK1 activation restores synaptic function under hypometabolic conditions. (**A**) Assay schematic: Cultured primary hippocampal neurons expressing vGlutI-pH or synapto-iATPSnFR2-miRFP670nano3 were incubated in imaging medium of interest for 5 min before 10 bouts of AP firing (100 APs at 10 Hz, highlighted as black bars in the traces) delivered every minute. Created using Biorender.com. (**B**) Ensemble average vGlutI-pH responses are normalized to the maximal sensor fluorescence revealed by perfusion with 50 mM NH_4_Cl. With sufficient fuel (5 mM glucose, teal), efficient SV recycling persists for all rounds of stimulation, but in low glucose (0.1 mM glucose, blue), SV recycling gradually slows, resulting in a net accumulation of fluorescence. (**C**) The ensemble average fraction of the fluorescence remaining 55 s after stimulation for each bout for high- and low-glucose conditions. Means ± SEM for the data shown in (B). 5 mM glucose, *n* = 5; 0.1 mM glucose, *n* = 23. *P < 0.05 and **P < 0.01, two-way analysis of variance (ANOVA). (**D**) Ensemble average vGlutI-pH fluorescence in neurons expressing PGK1-HALO (red) and Terazosin-treated (green) shows that synaptic endurance is restored compared to 0.1 mM glucose control (blue). (**E**) The remaining fluorescence 55 s after stimulation after each train is plotted. Means ± SEM. Control, *n* = 23; PGK1-HALO, *n* = 12. **P < 0.01 and ***P < 0.001, two-way ANOVA multiple comparisons to control. Terazosin, *n* = 10. ##*P* < 0.01 and ###*P* < 0.001, two-way ANOVA multiple comparisons to control. (**F**) PGK1 (magenta) and synapsin I/II (cyan) immunofluorescence shows that PGK1 is present in nerve terminals (white arrows). Scale bar, 6 μm.

To identify molecular components that might confer metabolic resilience, we devised a synaptic endurance test based on previously established synaptic vesicle (SV) recycling assays carried out in primary dissociated hippocampal neurons (Fig. 1A). Here, neurons are subjected to repeated bouts of stimulation at regular intervals. With sufficient fuel (5 mM glucose), SV recycling can be sustained through at least 10 rounds of action potential (AP) bursts delivered at minute intervals as measured with vGlut-pHluorin (vGlut-pH) fluorescence (Fig. 1, B and C). This synaptic endurance fails when extracellular glucose is lowered to 0.1 mM (Fig. 1, B and C), which is likely much lower than the concentration of glucose in vivo (*22*), as successive rounds of stimulation lead to a gradual slowing of the endocytic retrieval/reacidification process and a gradual increase in the poststimulus fluorescence.

We carried out a genetic expression suppressor screen of glycolytic enzymes to see whether any single one, when overexpressed, would allow synapses to sustain function under limited fuel availability. Of the enzymes evaluated (see Materials and Methods and fig. S1, A and B), only one, PGK1, conferred significant hypometabolic resilience. PGK1, when overexpressed, was able to fully support synaptic function in 0.1 mM glucose, exhibiting robust SV recycling for all 10 rounds of activity tested (Fig. 1, D and E, and fig. S1, A and B).

Quantitative immunofluorescence for PGK1 and synapsin showed that native PGK1, although expressed throughout the cell, was highly concentrated in nerve terminals (Fig. 1F and fig. S1, C and D). The gene encoding PGK1 is located on the X chromosome, suggesting that expression of PGK1 might differ in male versus female neurons. To test this hypothesis, we cultured primary neurons separately from male and female littermates (see Materials and Methods). Quantitative immunofluorescence for PGK1 in synapsin-positive nerve terminals, however, failed to reveal any difference in the abundance of this enzyme at nerve terminals that was sex specific (fig. S1E), suggesting that X inactivation of the gene encoding PGK1 is complete. The functional rescue was conducted using a HALO-tagged PGK1 allowing us to visualize the expressed protein distribution in live cells, confirming that the functional rescue was neuron specific (fig. S1F). Comparison of quantitative immunocytochemistry for PGK1 and live-cell labeling of the HALO moiety showed that a twofold overexpression of PGK1 was sufficient to confer hypometabolic resilience (fig. S1, G to J). As expected, SV recycling was impaired in neurons expressing a short hairpin RNA (shRNA)–targeting PGK1 (fig. S2, A to D).

### TZ restores synaptic function under hypometabolic conditions

As TZ was a starting point for implicating a pivotal role for PGK1 in PD, we examined whether TZ would functionally mimic PGK1 expression in our synaptic endurance paradigm. Treating neurons with 10 μM TZ conferred significant resilience to stimulation in low glucose (Fig. 1, D and E), albeit to a lesser extent than with PGK1 expression (see Discussion). This impact was eliminated in neurons expressing an shRNA-targeting PGK1 (fig. S2, E and F). A modified version of TZ that lacks potency for TZ’s known clinical target, the a_1_ adrenergic receptor, TZ-md (*23*), was equally effective at conferring synaptic endurance (fig. S2, G and H).

### PGK1 expression accelerates local ATP production

**Fig. 2.**
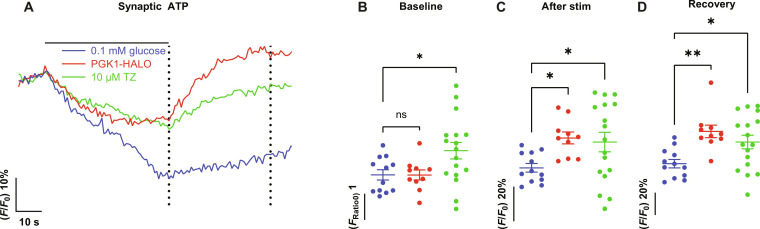
PGK1 activation locally accelerates synaptic ATP production. (**A**) Ensemble average synapto-iATPSnFR2-miRFP670nano3 traces for 0.1 mM glucose control (blue), PGK1-HALO (red), and TZ-treated (green) transfected cells stimulated with 600 APs at 10 Hz. PGK1-HALO and TZ neurons show a significant activity-dependent up-regulation of ATP synthesis following activity. Comparison of absolute nerve terminal ATP values (**B**) before stimulus and normalized values (**C**) at the end of the stimulus [left dotted line in (A)] and (**D**) 50 s after stimulus [right dotted line in (A)] in control, PGK1-HALO–expressing, and TZ-treated neurons. Means ± SEM. Control, *n* = 12; PGK1-HALO, *n* = 10; TZ, *n* = 17. *P < 0.05 and **P < 0.01, one-way ANOVA. ns, not significant.

The impact of PGK1 expression on hypometabolic synapse function strongly implies that increasing this enzyme’s abundance leads to a robust change in the kinetics of presynaptic ATP synthesis. To test this idea, we carried out measurements of presynaptic ATP dynamics using a next-generation genetically encoded ratiometric ATP reporter (synapto-iATPSnFR2-miRFP670nano3) targeted to nerve terminals (Fig. 1A), as well as a variant that is ATP insensitive (*24*). Under hypometabolic conditions, a 60-s burst of 600 APs led to a 30% depletion of presynaptic ATP recovering minimally in the next 50 s (Fig. 2, A, C, and D), while an ATP-insensitive sensor remained unresponsive (fig. S3, A to C). In neurons expressing PGK1-HALO, the initial prestimulus ATP values were unchanged (Fig. 2B); however, ATP decreased less during the stimulus (decreasing by 14%) (Fig. 2, A and C) and recovered rapidly over the next 30 s (Fig. 2, A and D). These data demonstrate that increased PGK1 abundance is sufficient to increase ATP production kinetics during demand, confirming that, during activity, PGK1 is a rate-limiting enzyme in nerve terminal glycolysis. Parallel ATP measurements showed that TZ also leads to improved nerve terminal ATP production. In neurons treated with TZ, resting ATP levels were slightly elevated (Fig. 2B), ATP depletion during strong stimulation in low glucose was blunted (Fig. 2, A and C), and the recovery following such a stimulus was accelerated compared to control (Fig. 2, A and D). As the end product of glycolysis, pyruvate, is the primary fuel for oxidative phosphorylation, we wondered whether the boost in ATP production mediated by PGK1 expression relied on mitochondrial function. To examine this, we measured the kinetics of ATP depletion in 5 mM glucose. In the presence of oligomycin, an inhibitor of the *F*_0_-*F*_1_ ATP synthase, presynaptic ATP levels become depleted by ~37% (fig. S3, D to F) during stimulation and only partially recover over the next 50 s. In contrast, in neurons expressing PGK1-HALO, the decline in presynaptic ATP is blunted (~15% drop), and ATP levels fully recover over the next 60 s (fig. S3, D to F). These data collectively support the concept that PGK1 under hypometabolic conditions is rate-limiting for ATP production at nerve terminals and that TZ increases PGK1 activity and thereby ATP production.

### PGK1 expression restores PARK20 synaptic dysfunction

**Fig. 3.**
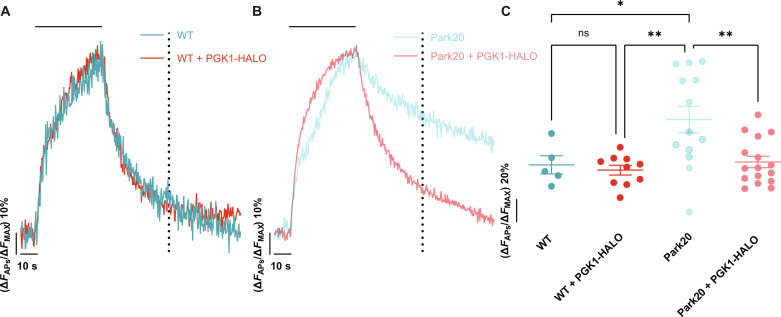
PGK1 expression restores PARK20 synaptic dysfunction. Ensemble average vGlutI-pH traces in primary WT (**A**) and PARK20/Synj1 R258Q knock-in (**B**) mouse cortical neurons stimulated with 600 APs (10 Hz, indicated by the black bar) in 5 mM glucose with PGK1-HALO expression (red) or without (teal). (**C**) Quantification of the remaining fluorescence of the vGlutI-pH traces 60 s after stimulation (indicated by the dotted line). Means ± SEM. WT, *n* = 5; WT + PGK1-HALO, *n* = 10; Park20, *n* = 13; Park20 + PGK1-HALO, *n* = 16. *P < 0.05 and **P < 0.01, one-way ANOVA.

Previous analysis of nerve terminal function in neurons derived from mice harboring different PARK mutations (PARK2, PARK7, PARK8, PARK19, and PARK20) revealed that deficits in synaptic recycling were common (*25*–*29*). We sought to determine whether PGK1 expression could reverse PD-driven SV recycling deficits. Nerve terminals derived from PARK20 mice, which harbor the R258Q mutation in Synj1, are characterized by synaptic abnormalities and impaired SV recycling (*29*). Expression of PGK1-HALO in wild-type (WT) neurons had minimal impact on SV recycling kinetics (Fig. 3, A and C). However, PGK1-HALO expression in PARK20 nerve terminals reversed the SV recycling deficit (Fig. 3, B and C). These experiments suggest that the PD driving mutation in Synj1 either creates a metabolic burden or impairs ATP production in a fashion that can be overcome by PGK1 overexpression.

### PARK7/DJ-1 is necessary for axonal PGK1 activity

**Fig. 4.**
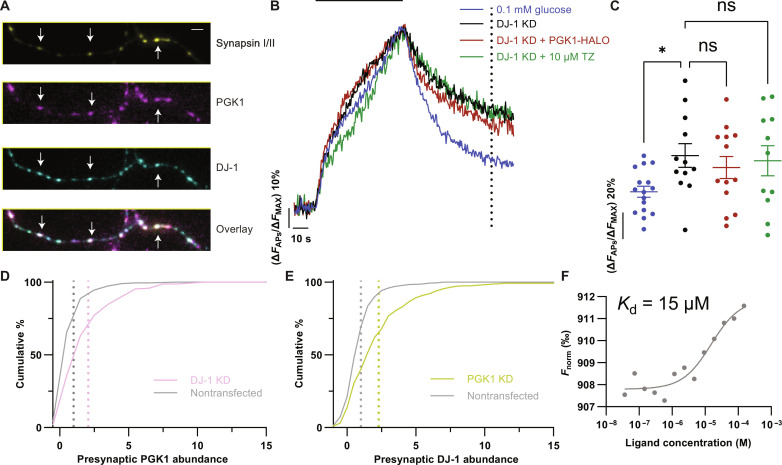
PARK7/DJ-1 is necessary for axonal PGK1 activity. (**A**) Immunostaining against PGK1 (magenta), synapsin I/II (yellow), and DJ-1 (cyan) shows that DJ-1 and PGK1 are both present in nerve terminals (white arrows). Scale bar, 2 μm. Created using Biorender.com (**B**) KD of DJ-1 (black) slows SV endocytosis kinetics compared to 0.1 mM glucose control (blue) vGlutI-pH–transfected cells, when neurons were challenged with 600 APs delivered at 10 Hz (black bar) . PGK1-HALO expression (red) or TZ incubation (green) failed to restore SV kinetics. (**C**) Quantification of the remaining fluorescence 60 s after stimulation [dotted line in (B)]. Means ± SEM. Control, *n* = 16; DJ-1 KD, *n* = 12; DJ-1 KD + PGK1-HALO, *n* = 13; DJ-1 KD + 10 μM TZ, *n* = 11; (ns) *P* > 0.05 and *P < 0.05, one-way ANOVA. (**D**) Cumulative histogram of presynaptic PGK1 fluorescence immunostaining intensity in control (gray) and DJ-1 KD nerve terminals (pink). DJ-1 KD, *n* = 171; nontransfected, *n* = 500. (**E**) DJ-1 fluorescence immunostaining in control (gray) and PGK1 KD nerve terminals (yellow). PGK1 KD, *n* = 289; nontransfected, *n* = 500. Distributions in both KDs are significantly different from their respective controls. *P* < 0.001, Kolmogorov-Smirnoff test. (**F**) MST identified an in vitro interaction between PGK1 and DJ-1 with a low micromolar affinity (~15 μM).

DJ-1 is a chaperone with strong structurally similarity to heat shock protein 31 (*30*) and is the product of the PARK7 gene, a genetic driver of familial PD (*31*, *32*). The precise DJ-1 clientele related to PD is poorly understood (*33*), but loss of DJ-1 also leads to impaired SV recycling (*25*). DJ-1 shows good presynaptic localization (Fig. 4A), and, as with DJ-1 knockout neurons, depletion of DJ-1 via shRNA-mediated knockdown (KD) (fig. S3, G and H), mimicking loss-of-function PARK7 mutations, impaired SV recycling following a large stimulus (Fig. 4, B and C) that could be reversed by reexpression of an shRNA-insensitive variant, but not one in which the catalytic cysteine of DJ-1 was mutated (C106A) (fig. S3, I and J). However, unlike with PARK20-driven SV recycling deficits, neither expression of PGK1 nor TZ was able to restore SV recycling kinetics in DJ-1 KD synapses (Fig. 4, B and C), and the PGK1/TZ rescue of hypometabolic conditions were abolished in absence of DJ-1 (fig. S4, A to D). To determine whether loss of DJ-1 affected PGK1 expression, we carried out quantitative immunostaining against nerve terminal PGK1 in DJ-1 KD neurons. These experiments demonstrated that contrary to what would be predicted from a simple proteostatic role of DJ-1, presynaptic PGK1 levels increased ~2-fold when DJ-1 was depleted (Fig. 4D). The increase in PGK1 abundance suggests that a feedback loop sensing PGK1 activity may be responding to the lack of DJ-1. When we depleted PGK1 in neurons (via shRNA-mediated KD), quantitative immunostaining against nerve terminal DJ-1 shows that its abundance is also up-regulated ~2-fold (Fig. 4E). This reciprocal relationship strongly implies a close functional relationship between these two proteins. Consistent with previous measurements, we showed using purified recombinant proteins that DJ-1 can pull down PGK1 in human embryonic kidney–293T cells and in vitro (fig. S4, E and F), and using microscale thermophoresis (MST), we documented a weak in vitro interaction (Fig. 4F), consistent with a chaperone-client interaction.

### PARK7/DJ-1 is necessary for PGK1-mediated synaptic resilience

**Fig. 5.**
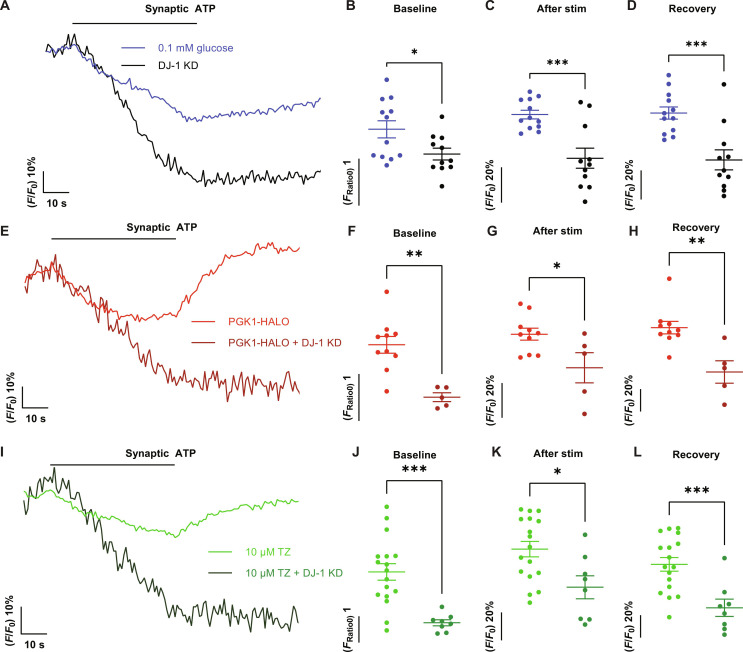
PARK7/DJ-1 is required for PGK1-mediated synaptic resilience. (**A**) The kinetics of presynaptic ATP normalized to the prestimulus values when neurons were stimulated with 600 APs at 10 Hz in 0.1 mM glucose (blue) and in the absence of DJ-1 (black) reveal a significantly larger activity-induced drop in ATP and defective poststimulus recovery. Comparison of absolute nerve terminal ATP values before stimulus (**B**) and normalized values at the end of the stimulus (**C**) and 50 s after stimulus (**D**) in control and DJ-1 KD neurons. Means ± SEM. Control, *n* = 12; DJ-1 KD, *n* = 11. *P < 0.05 and ***P < 0.001, unpaired *t* test. (**E**) The kinetics of presynaptic ATP normalized to the prestimulus values when neurons were stimulated with 600 APs at 10 Hz in 0.1 mM glucose with PGK1-HALO and DJ-1 KD (dark red) reveal an inability of PGK1 to accelerate ATP kinetics in absence of DJ-1. Comparison of absolute nerve terminal ATP values before stimulus (**F**) and normalized values at the end of the stimulus (**G**) and 50 s after stimulus (**H**) in PGK1-HALO with and without DJ-1 KD neurons. Means ± SEM. PGK1-HALO, *n* = 10; PGK1-HALO + DJ-1 KD, *n* = 5. *P < 0.05 and **P < 0.01, unpaired *t* test. (**I**) The kinetics of presynaptic ATP normalized to the prestimulus values when neurons were stimulated with 600 APs at 10 Hz in 0.1 mM glucose in TZ and DJ-1 KD (dark green) reveal an inability of TZ to accelerate ATP kinetics in absence of DJ-1. Comparison of absolute nerve terminal ATP values before stimulus (**J**) and normalized values at the end of the stimulus (**K**) and 50 s after stimulus (**L**) in TZ and TZ with DJ-1 KD neurons. Means ± SEM. TZ, *n* = 17; TZ + DJ-1 KD, *n* = 8. *P < 0.05 and ***P < 0.001, unpaired *t* test.

The inability of PGK1 expression or TZ to rescue hypometabolic function in a DJ-1 KD, together with the reciprocal response to depletion of PGK1 and DJ-1, implies that DJ-1 might control ATP production during activity. To test this idea, we carried out measurements of presynaptic ATP dynamics in DJ-1 KD neurons using synapto-iATPSnFR2-miRFP670nano3. These experiments revealed an inability of DJ-1 KD nerve terminals to sustain ATP production during stimulation under hypometabolic conditions (Fig. 5A), showing a 60% drop in ATP, more than double that in control neurons (Fig. 5C), and exhibited no recovery thereafter (Fig. 5D), while also exhibiting diminished baseline ATP (Fig. 5B). Consistent with the inability of PGK1 activity enhancement to rescue hypometabolic synapse function, both PGK1 overexpression (Fig. 5, E to H) and TZ-mediated (Fig. 5, I to L) ATP acceleration were impaired following stimulation in DJ-1 KD neurons. The impaired ATP production resulting from loss of DJ-1 was due to a loss of glycolytic function and not mitochondrial ATP production as slowed SV recycling in low glucose was only apparent when we additionally blocked mitochondrial ATP production (fig. S4, G, H, and J). Furthermore, when glucose was substituted with a mixture of lactate and pyruvate, SV recycling in DJ-1 KD neurons was indistinguishable from control (fig. S4, I and J). Thus, DJ-1 is necessary for PGK1 function when fuel becomes limiting, suggesting that PGK1 is a bona fide client of DJ-1 chaperone activity.

### PGK1 expression protects against dopaminergic axon degeneration in the striatum

**Fig. 6.**
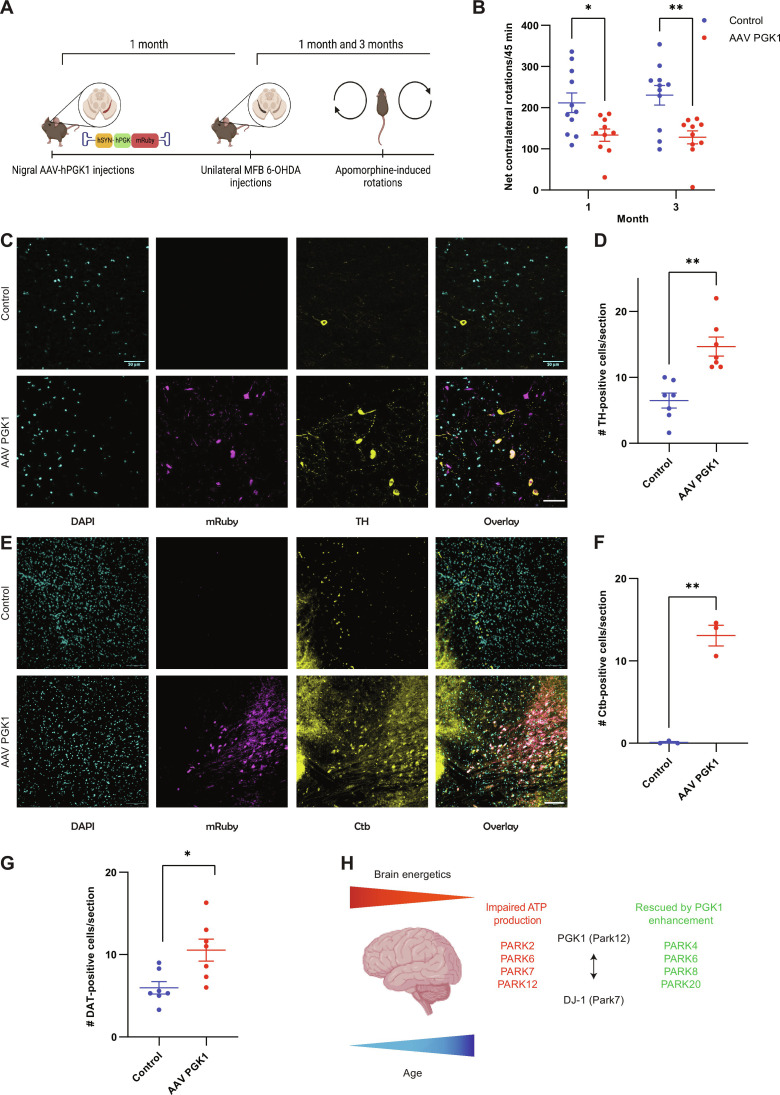
PGK1 expression in vivo protects against dopaminergic axon degeneration. (**A**) An AAV-driving hSyn PGK1-mRuby was injected unilaterally in the substantia nigra 30 days before 6-OHDA was injected in the MFB, with control mice only receiving the 6-OHDA injections. At 30 or 90 days after the 6-OHDA injection, mice were subjected to apomoprhine-induced rotation tests. (**B**) PGK1-mRuby expression significantly suppressed the apomorphine-induced rotations. Means ± SEM. Control, *n* = 11; AAV PGK1, *n* = 10. *P < 0.05 and **P < 0.01, two-way ANOVA. (**C**) Immunostaining of control and AAV PGK1 mid-brain brain slices against 4′,6-diamidino-2-phenylindole (DAPI), mRuby, and TH. Scale bars, 50 μm. (**D**) Quantification of the number of TH-positive cells showed a significant increase in animals receiving the PGK1 AAV. Control, *n* = 7; AAV PGK1, *n* = 7. **P < 0.01, unpaired *t* test. (**E**) Before immunostaining, the striata of the animals were injected with the retrograde tracer cholera toxin subunit b (Ctb). Scale bars, 100 μm. (**F**) Quantification of the number of Ctb-positive cells also showed a significant increase after lesion in case of the PGK1 AAV. Control, *n* = 3; AAV PGK1, *n* = 3. **P < 0.01, unpaired *t* test. (**G**) Quantification of the number of DAT-positive cells showed a significant increase in in animals receiving the PGK1 AAV. Control, *n* = 7; AAV PGK1, *n* = 7. *P < 0.05, unpaired *t* test. (**H**) Human brain energetics decline physiologically with age, one of the most important risk factors in PD. Several PARK animal models (red) exhibit deficits in energy homeostasis. Up-regulating PGK1 activity (part of PARK12) and its necessary partner PARK7/DJ-1 has now been shown to rescue at least four different PARK genes (green), proposing a crucial unifying theme of neuronal hypometabolism in PD pathology. (H) created using Biorender.com.

Previous in vitro analysis demonstrated that TZ could only modestly accelerate PGK1 activity (*10*), casting doubt on whether the impact of TZ on PD in both human (*12*) and animal models (*11*) is attributable to PGK1 enhancement. To determine whether increasing PGK1 expression could provide in vivo protection, 30 days before a unilateral 6-hydroxy-DA (6-OHDA) injection in the medial forebrain bundle (MFB), an adeno-associated virus (AAV) driving neuronal expression of PGK1-mRuby was injected into the SNc on the same side of the brain that will later receive the 6-OHDA injection (Fig. 6A). 6-OHDA injection leads to PD-resembling axonal degeneration and can be assessed by application of DA receptor (D2R) agonists. In control [phosphate-buffered saline (PBS) injected in SNc, 6-OHDA–lesioned] mice, application of the D2R agonist apomorphine led to a robust inducement of whole-body rotations measured 1 or 3 months after the 6-OHDA injection. This rotation phenotype was significantly suppressed in animals that received the PGK1 AAV injection (Fig. 6B). Although the precise mechanism of 6-OHDA–driven axon loss is debated, recent experiments support the idea that degeneration begins in the axonal projections (*34*). Retrospective histology carried out 90 days after the 6-OHDA injection showed that the number of tyrosine hydroxylase (TH)–positive (Fig. 6, C and D) and DA transporter antibody (DAT)–positive (Fig. 6G and fig. S5A) neurons in the SNc was ~2.5-fold higher in animals that received the AAV harboring PGK1 and that most of these DA neurons also stained positive for tagged PGK1 (Fig. 6C and fig. S5A). Striatal injections of a retrograde tracer showed that in addition to promoting survival of SNc cell somas, PGK1 expression preserved SNc-striatal axonal projections (Fig. 6, E and F), demonstrating that PGK1 expression protects the axonal integrity of dopaminergic neurons.

## DISCUSSION

It has long been suspected that neuronal susceptibility in PD results from a combination of a general age-related decline in the ability to adequately fuel the brain, the outsized vulnerability of highly arborized and energetically demanding SNc DA neurons, and genetic lesions that impair either the efficacy of ATP production or its use within the brain. To date, human genetic studies have led to the identification of at least 23 drivers of familial PD, the PARK genes (*35*). Although two of the PARK genes, PARK2 (PARKIN) and PARK6 (PINK1), are potentially directly linked to bioenergetics as they participate in mitochondrial quality control (*5*), the connection between other PARK genes and bioenergetics is less clear. The discovery that TZ is both therapeutically protective in human (*12*, *14*) and numerous animal models (*11*) provided compelling evidence that bioenergetic dysfunction might be central to PD. One challenge in interpreting these findings is whether the protective impact of TZ was really attributable to an enhancement of PGK1 activity, since the in vitro data only showed a very modest impact of TZ on PGK1 function (*10*). Our data speak directly to this and demonstrate both that enhancing PGK1 activity protects striatal DA axons in vivo and accelerates the kinetics of subcellular ATP production in vitro. Our experiments with TZ were in broad agreement with the original data describing TZ’s neuroprotective effects (*11*).

Our data reveal an unexpected link between PGK1 and PD, as the ability of increased PGK1 abundance to accelerate ATP production was completely dependent on PARK7/DJ-1, a known genetic driver of PD in humans. Although an alternate interpretation is that DJ-1 prevents deleterious modification of several glycolytic enzymes, as was recently proposed (*36*), our data are consistent with the idea that there is a functional partnership between DJ-1 and PGK1, whereby DJ-1 directly modulates PGK1 activity. This interpretation is supported both by our in vitro binding data and by the apparent cross-regulation of the abundance of these two proteins. We propose that rather than acting as a classical chaperone to simply restore proper client folding and preventing protein degradation, DJ-1 instead acts to sustain enzyme activity.

Previous studies showed that TZ was remarkably protective in several genetic models of PD (*11*), including PARK4, PARK6, and PARK8. However, the interpretation of these data hinged upon the previous identification of the off-target binding of TZ to PGK1. The in vitro analysis in that work reported that TZ could accelerate PGK1 activity, but only by ~4% (*10*), casting doubt on whether the in vivo impact of TZ was mediated by PGK1. Our studies unambiguously show that enhancing PGK1 activity is highly protective and complements previous findings by additionally demonstrating that specific synaptic deficits driven by PARK20/Synj1 R258Q can also be reversed by PGK1 expression. These data all imply that a common underlying link between the many disparate genetic drivers of PD is likely bioenergetic in origin. We anticipate (Fig. 6H) that certain mutations compromise ATP production, either, for example, by impairing mitochondrial function as is implied in PARK2/PARK6 or by impairing glycolysis as seems to be occurring with PARK7/DJ-1 and potentially PARK12. Other drivers of PD may result in creating undue metabolic burdens, which, in turn, compromise axon and synapse function, as may be the case for PARK20/Synj1.

It is notable that overexpression of PGK1, unlike TZ, did not affect resting ATP levels (Fig. 2B). These suggest that what is critical to provide protection is not so much the absolute levels of ATP but, instead, the ability to synthesize ATP quickly, when and where it is needed. Thus, the relevant biophysical parameter may really be the need to generate a sufficient flux of ATP, suggesting that certain biological processes become irretrievably impaired when ATP cannot be hydrolyzed in this process within a certain time frame.

These results establish PGK1 as a crucial leverage point in synaptic function and PD-related dysfunction. Therefore, the mechanisms that control PGK1 abundance, activity, and localization, which are not well understood, are of significant therapeutic potential. In humans, PGK1 mutations present usually in three broad clinical deficits characterized by symptoms associated with anemia, myopathy, and neurological dysfunction (*37*). This strongly suggests that different cells and tissues are differentially vulnerable to different mutations, implying that their local regulation may differ. Further investigation into how local abundance and activity are controlled in different tissues should prove insightful. Our study significantly strengthens the case that PGK1 biology is of direct therapeutic interest for PD and neurodegenerative diseases in general. It is notable that in a mouse model of spinal muscular atrophy, Boyd and colleagues (*21*) found a strong correlation between PGK1 expression and the resilience to spinal cord motor neuron degeneration and that overexpression of PGK1 was strongly protective in a zebrafish model of this genetic disease. Similarly, PGK1 expression in *Drosophila* neurons, but not in muscle, protected flies from rotenone-induced motor neuron dysfunction (*11*). A detailed understanding of the molecular mechanism underlying PGK1 activation will have the potential to open new therapeutic routes for this disease.

## MATERIALS AND METHODS

All protocols for this study have been deposited at dx.doi.org/10.17504/protocols.io.3byl495nzgo5/v1.

### Reagents

Chemical reagents were purchased from MilliporeSigma. TZ was purchased from Tocris Bioscience (1506). TZ-md was purchased from LGC (TRC-T105020). Oligomycin was purchased from MilliporeSigma (O4876). Antibodies used are anti (a)-PGK1 (Abcam, ab199438; 1:1000), a-DJ-1 (Cell Signaling Technology, 5933, RRID: AB_11179085; 1:250; and Santa Cruz Biotechnology, sc-55572, RRID: AB_831639; 1:250), a-synapsin I/II (Synaptic Systems, 106 004, RRID: AB_1106784; 1:1000), a–glyceraldehyde-3-phosphate dehydrogenase (GAPDH) (Cell Signaling Technology, 5174, RRID: AB_10622025; 1:1000), a–tubulin-βIII (R&D Systems, MAB1195, RRID: AB_357520; 1:1000), a–green fluorescent protein (GFP) (Thermo Fisher Scientific, A10262, RRID: AB_2534023; 1:500), a-TH (Millipore, AB1542, RRID: AB_90755; 1:1000), a-DAT (Proteintech, 22524-1-AP, RRID: AB_2879116; 1:100), a-Myc (Cell Signaling Technology, 2278, RRID: AB_490778), a–hemagglutinin (HA) (BioLegend, 901501, RRID: AB_2565006), a-His (Cell Signaling Technology, 2365, RRID: AB_2115720), and a-TagFP nanobody conjugated with ATTO488 (NanoTag Biotechnologies, N0502-At488-L, RRID: AB_2744623; 1:500). Alexa Fluor–conjugated fluorescent secondary antibodies were obtained from Life Technologies and used at 1:500.

### Animals

All animal-related experiments were performed in accordance with protocols approved by the Weill Cornell Medicine Institutional Animal Care and Use Committee, protocols numbers 0601-450A and 2009-0026. WT rats were of the Sprague-Dawley strain (Charles River Laboratories, strain code: 400, RRID: RGD_734476). The PARK20/Synj1 R258Q knock-in mice (*29*) were maintained in the De Camilli laboratory. For behavioral experiments, male C57BL/6J mice (the Jackson Laboratory, strain #000664, RRID: MSR_JAX:000664) were housed two to five per cage and kept at 22°C on a reverse 11 a.m. light/11 p.m. dark cycle, with standard mouse chow and water provided ad libitum throughout the duration of the study.

### Plasmids

The following previously published DNA constructs were used: vGLUT1-pH (*38*), DJ-1 (a gift from M. Cookson, Addgene, plasmid #29347, RRID: Addgene_29347, http://n2t.net/addgene:29347), synapto-iATPSnFR2-miRFP670nano3 (Addgene, plasmid #209730, RRID: Addgene_209730, http://n2t.net/addgene:209730), and synapto-cpsfGFP-miRFP670nano3 (Addgene, plasmid #214926, RRID: Addgene_214926, http://n2t.net/addgene:214926). For this study, these new plasmids were generated: PLKO mTagBFP2 (Addgene, plasmid# 191566, RRID: Addgene_191566, http://n2t.net/addgene:191566), PGK1 KD (targeting sequence GCTAAGCAGATTGTTTGGAAT, Addgene, plasmid #222869, RRID: Addgene_222869, http://n2t.net/addgene:222869), DJ-1 KD (targeting sequence ATCTGGGTGCACAGAACTTAT, Addgene, plasmid #222870, RRID: Addgene_222870, http://n2t.net/addgene:222870), PGK1-HALO (Addgene, plasmid #220910, RRID: Addgene_220910, http://n2t.net/addgene:220910), AAV PGK1-mRuby (Addgene, plasmid #220911, RRID: Addgene_220911, http://n2t.net/addgene:220911), DJ-1 C106A (Addgene, plasmid #220914, RRID: Addgene_220914, http://n2t.net/addgene:220914), PGK1-HA (Addgene, plasmid #220913, RRID: Addgene_220913, http://n2t.net/addgene:220913), hSyn hexokinase 1 (Addgene, plasmid #220915, RRID: Addgene_220915, http://n2t.net/addgene:220915), hSyn alodolase A (Addgene, plasmid #220916, RRID: Addgene_220916, http://n2t.net/addgene:220916), hSyn alodolase C (Addgene, plasmid #220917, RRID: Addgene_220917, http://n2t.net/addgene:220917), hSyn GAPDH (Addgene, plasmid #220918, RRID: Addgene_220918, http://n2t.net/addgene:220918), hSyn PGK1 (Addgene, plasmid #220919, RRID: Addgene_220919, http://n2t.net/addgene:220919), and hSyn pyruvate kinase M1 (Addgene, plasmid #220920, RRID: Addgene_220920, http://n2t.net/addgene:220920).

### Primary neuronal culture

Primary neuronal culture and transfection were applied as described (*39*). For Park20 experiments, WT and Park20 homozygous littermates were used from a WT/Park20 heterozygous cross.

### Animal genotyping

For genotyping the PARK20 mouse litters and the sex-specific WT Sprague-Dawley rat litters, first DNA was extracted from tail clips using the commercial kit Platinum Direct PCR Universal Master Mix (Thermo Fisher Scientific, A44647100). The DNA was then subjected to polymerase chain reaction (PCR) using components from the same kit and the published primers for PARK20 mice (*29*) or sex male/female rats (*40*). PCR products were then resolved in a 1.5% standard agarose DNA gel.

### Live-cell imaging

Live-cell imaging was performed as described (*41*). Neurons were continuously perfused at 0.1 ml/min with a Tyrode’s solution containing 5 mM glucose, 50 mM Hepes, 119 mM NaCl, 2.5 mM KCl, 2 mM CaCl_2_, 2 mM MgCl_2_, 0.01 mM 6-cyano-7-nitroquinoxaline-2,3-dione, and 0.05 mM d,l-2-amino-5-phospho-novaleric acid (pH 7.4) for high-glucose experiments. For low glucose, the Tyrode’s buffer used contained 0.1 mM glucose, 54.9 mM Hepes, 119 mM NaCl, 2.5 mM KCl, 2 mM CaCl_2_, 2 mM MgCl_2_, 0.01 mM 6-cyano-7-nitroquinoxaline-2,3-dione, and 0.05 mM d,l-2-amino-5-phospho-novaleric acid (pH 7.4). When oligomycin was used, it was added at 2 μM (MilliporeSigma, O4876). For vGlutI-pH normalization, cells were perfused with Tyrode’s solution containing 50 mM NH_4_Cl (pH 7.4). For TZ experiments, TZ preincubated with cells for 12 to 16 hours at a final concentration of 10 μM. For all experiments due to the hypometabolic aspect of most assays, only one neuron was imaged per individual cultured coverslip, so *N* equals both individual neuron and coverslip.

### Genetic expression suppressor screen

Primary hippocampal neurons cotransfected with vGlutI-pH and a plasmid encoding one of the following glycolytic enzymes: hexokinase 1, GAPDH, PGK1, alodolase A, aldolase C, or pyruvate kinase M1. Neurons were incubated in imaging medium containing 0.1 mM glucose for 5 min after mounting into the stimulus chamber. Neurons were then challenged with 10 bouts of AP firing (100 APs at 10 Hz) delivered every minute. Cells were lastly perfused with imaging medium containing 50 mM NH_4_Cl.

### vGlutI-pH analysis

For vGlutI-pH experiments, nerve terminals responding to the first train of APs were selected and background-subtracted. In case of the repeated stimulation assay, the traces are reported as Δ*F* by subtracting the initial fluorescence signal before stimulation and normalized to total sensor fluorescence, revealed by the 50 mM NH_4_Cl Tyrode’s solution, yielding Δ*F*_APs_/Δ*F*_NH_4__. For single-train experiments, the traces are reported Δ*F*_APs_/Δ*F*_MAX_, by normalizing to the fluorescence peak during the AP train. The remaining fluorescence measurements are calculated at the specified time point for each dataset. All graphs were made with GraphPad Prism 10.0.2 (RRID: SCR_002798, www.graphpad.com/) and sketches with BioRender (RRID: SCR_018361, http://biorender.com/).

### Synapto-iATPSnFR2-miRFP670nano3 analysis

For synapto-iATPSnFR2-miRFP670nano3 experiments, nerve terminals were selected in the miRFP670nano3 channels, blind to the iATPSnFR2 channel, and then background-subtracted. The traces are reported as iATPSnFR2-to-miRFP670nano3 ratio, *F*_Ratio_. Single-cell data were filtered by removing any cells that exhibited no change to activity.

### Immunocytochemistry

For immunostaining experiments, we used a modified culture protocol to seed cells in a lower density. These cells were instead plated on poly-d-lysine–treated coverslips in absence of cylinders and transfected using a Lipofectamine 2000 reagent, as previously described (*42*). Cells on a coverslip were fixed using 4% paraformaldehyde (PFA), treated with 50 mM NH_4_Cl, and then permeabilized using 0.1% Triton X-100. Cells were blocked using a 5% bovine serum albumin buffer, and the antibodies were incubated in the same buffer. Last, cells were mounted on glass slides using ProLong Diamond (Thermo Fisher Scientific, P36970). For shRNA quantification, regions of interested including the cell somas were selected. For presynaptic analysis, regions of interested that were synapsin I/II–positive/negative were selected blindly to the other channels.

### AAV craniotomy and in vivo PD model

Stereotactic surgical procedures were performed on 8- to 12-week-old mice, weighing 20 to 25 g, under a mixture of ketamine/xylazine anesthesia. Ketamine (Butler Animal Health Supply) and xylazine (Lloyd Laboratories) were administered at concentrations of 110 and 10 mg/kg of body weight, intraperitoneally (ip), respectively. After the induction of anesthesia, the animals were placed into a stereotactic frame (David Kopf Instruments). All AAV infusions were performed using a 10-μl stereotactic syringe attached to a microinfusion pump (World Precision Instruments) at a rate of 0.1 to 0.4 μl/min. To prevent reflux, after each infusion, the injection needle was left in place for 5 min, withdrawn a short distance (0.3 to 0.5 mm), and then left in the new position for an additional 2 min before removal.

hSyn hPGK1-mRuby AAV or PBS was injected unilaterally into the mouse SNc (anterior-posterior, −3.0 mm; medial-lateral, ±1.2 mm; dorsal-ventral, −4.5 mm from bregma). All craniotomies were performed at a rate of 0.4 μl /min using a 10-μl World Precision Instruments syringe with a 33-gauge needle. A total of 2 × 10^11^ genomic particles/μl (3 μl in PBS) of vector was injected in test animals. Controls received 3 μl in PBS, which was used as a control to avoid potential confounding effects of a control AAV expressing a marker gene that could induce or potential toxicity and lead to a false conclusion of PGK-mediated neuroprotection. The mice were allowed a 6- to 8-week recovery before being subjected to the behavioral tests during which maximal transgene expression from AAV vectors is achieved. At the conclusion of the experiments, injection site accuracy within the targeted brain region was determined by immunohistochemistry for the specific transgene expressed by the AAV vector, and mice with mistargeted injections were excluded from analysis before their data were unblinded.

To generate 6-OHDA–lesioned mice, animals were injected with a total volume of 0.6 μl of 6-OHDA hydrobromide (Sigma-Aldrich) in PBS with 0.1% ascorbate unilaterally into the MFB at a concentration of 2.5 mg/ml and an infusion rate of 0.1 μl/min with a 10-μl Hamilton syringe with a 30-gauge needle. The coordinates for the injection were an anterior-posterior of −1.1 mm, a medial-lateral of −1.1 mm, a dorsal-ventral of −5.0 mm relative to bregma and the dural surface. Before lesion surgery, the norepinephrine reuptake inhibitor desipramine (25 mg/kg, ip) was administered via intraperitoneal injection at least 30 min before 6-OHDA infusion to protect neostriatal and cerebellar noradrenergic neurons from the toxin-induced damage. Mice were allowed 4 weeks to recover before being subjected to the apomorphine-induced behavioral test to estimate the extent of DA depletion in the substantia nigra (see following section). For striatal injections of CTB Alexa Fluor–conjugated 488 (Invitrogen, catalog no. C34775), unilateral injections were performed into the ipsilateral dorsal striatum to the 6-OHDA lesion side (anterior-posterior, +0.5 mm; medial-lateral, ±2.3 mm; dorsal-ventral, −3.5 mm from bregma) 1 week before euthanizing the animals.

### Apomorphine-induced rotation assay

All behavioral tests were run during the dark phase of the mouse daily cycle. The experiments were performed by an examiner blinded to treatment groups. To test for 6-OHDA lesion efficiency, rotational behaviors were performed. Briefly, mice were placed in a body harness connected to a transducer/swivel in a bowl-shaped testing arena (rotometer). Full 360° clockwise and counterclockwise rotations were measured over a 45-min period after drug injection for apomorphine-induced rotations. Apomorphine (Sigma-Aldrich) was dissolved in saline solution, 0.9% NaCl, and 0.1% ascorbate and administered at a dose of 0.25 mg/kg via subcutaneous injection.

EthoVision XT14 (RRID: SCR_000441, www.noldus.com/ethovision) was used to analyze offline rotational behaviors. The automatic animal detection with deep learning settings was used to track center-to-nose and center-to-tail points in defined arenas. Video recordings were used to analyze the following locomotion and rotational parameters: (i) distance traveled, (ii) activity percentage (%), and (iii) rotation >90° clockwise and counterclockwise. Tracking data were graphed with EthoVision analysis tools.

### Immunohistochemistry

On completion of all assessments, mice were deeply anesthetized with sodium pentobarbital (150 mg/kg, ip) and transcardially perfused with 4% PFA. Brains were extracted and postfixed overnight in 4% PFA, cryoprotected in 30% sucrose, and cut into 30-μm sections using a vibratome (Leica Microsystems). Free-floating sections were treated with antibodies to visualize proteins of interest using immunofluorescence labeling, including TH and DAT to quantify the extent of nigral neurodegeneration and mRuby fluorescence to estimate the transduction efficiency from our AAV vector.

Images were taken with an epifluorescence microscope (Olympus BX61 fluorescence microscope fitted with an Olympus DP71 digital camera) or a confocal microscope (Zeiss LSM 900) and analyzed with ImageJ software [version 1.52p, National Institutes of Health (NIH), USA, RRID: SCR_003070, https://imagej.net/). For quantification of TH-positive cells, coronal sections were sampled at intervals of 90 to 120 μm for immunostaining. Three nigral sections per mouse were identified using the mouse brain atlas of Paxinos (2007) and scanned bilaterally using a 20× objective. For each selected section, three randomly chosen regions of interest with fixed areas were selected for quantification. A Macro plugin was applied to each section obtained, with a Gaussian blur filter threshold set to 30 and a size filter to 20 to remove small objects and low-signal cells. TH-positive neurons were determined only when clear colocalization with 4′,6-diamidino-2-phenylindole (DAPI) staining was observed. Automatized detection using ImageJ software in seven mice per condition (controls versus treated).

### Microscale thermophoresis

His-tagged SUMO-PGK1 and DJ-1 full-length proteins were expressed in BL21 (DE3) *Escherichia coli* cells. One liter of cultures in LB medium with kanamycin (50 μg/ml) were grown at 37°C to optical density at 600 nm of 0.6 before induction with 0.7 mM isopropyl-β-d-1-thiogalactopyranoside, followed by incubation at 22°C for 18 hours. Cells were harvested by centrifugation at 4000 rpm for 20 min and frozen at −80°C. Thawed cell pellets were lysed by sonication on ice in 200 ml of lysis buffer [50 mM tris-HCl (pH 8.0), 350 mM NaCl, 1 mM phenylmethylsulfonyl fluoride, and 5 mM β-mercaptoethanol]. Lysates were clarified by centrifugation at 18,000 rpm for 45 min. Supernatants containing soluble proteins were applied to a 5-ml Ni–nitrilotriacetic acid (NTA)–agarose gravity column (QIAGEN) preequilibrated with lysis buffer. Bound proteins were washed with 200 ml of wash buffer (lysis buffer with 30 mM imidazole) and eluted in 30 ml of elution buffer (lysis buffer containing 150 mM NaCl and 250 mM imidazole). The eluted fractions were analyzed by SDS–polyacrylamide gel electrophoresis (PAGE), and protein concentrations were estimated by ultraviolet absorption at 280 nm. His-tagged DJ-1 protein fractions were pooled and dialyzed using dialysis tubing with a molecular weight cutoff (MWCO) of 3.5 kDa to eliminate imidazole and concentrated using an Amicon Ultra-50 centrifugal filter unit (MWCO, 10 kDa). His-SUMO-PGK1 was pooled and dialyzed with an MWCO of 3.5 kDa in 4 liter of SUMO cleavage buffer [50 mM tris-HCl (pH 7.5), 150 mM NaCl, and 0.2 mM tris(2-carboxyethyl)phosphine (TCEP)] at 4°C for 24 hours. SUMO protease was added, and the mixture was rocked slowly at 4°C for 24 hours. Cleavage efficiency was monitored at various time points using SDS-PAGE. The fully cleaved mixture was loaded onto a 5-ml Ni-NTA–agarose column equilibrated with binding buffer without imidazole [50 mM tris-HCl (pH 7.5), 150 mM NaCl, and 0.2 mM TCEP] and eluted as described above. The flow-through containing cleaved PGK1 was collected. Fractions containing untagged PGK1 protein were concentrated using an Amicon Ultra-50 centrifugal filter unit (MWCO, 30 kDa).

Purified His-tagged DJ-1 and untagged PGK1 proteins were subjected to further purification using gel filtration with a Superdex200 Increase 10/300 GL size exclusion column (GE Healthcare) on an AKTA FPLC system (Cytiva). The column was equilibrated and samples were run in buffer [20 mM sodium phosphate (pH 7.4) and 0.2 mM TCEP] at a flow rate of 0.4 ml/min at 4°C. Both proteins eluted at the expected volume based on their molecular weights. Peak fractions were collected and analyzed by Coomassie-stained SDS-PAGE. Protein containing fractions were pooled before subsequent MST analysis. Protein structural integrity was verified using circular dichroism.

To assess the interaction of PGK1 with DJ-1, His-DJ-1 was first labeled with RED-tris-NTA by mixing 100 μl of 200 nM His-DJ-1 with 100 μl of 100 nM RED-tris-NTA in PBS with Tween 20 (PBS-T) buffer and incubating for 30 min at room temperature in the dark. PGK1 was prepared in PBS-T buffer at 152 μM concentration. This stock solution was used for a 16-step 1:1 serial dilution in PBS-T buffer with 10-μl final volume in each sample, followed by addition of 10 μl of 100 nM RED-tris-NTA–labeled DJ-1 to each sample. After mixing by pipetting up and down, reactions were incubated at room temperature, in the dark, for 30 min and centrifuged at 14,000 rpm for 10 min at 4°C before loading into Monolith NT.115 premium capillaries (NanoTemper). Samples were loaded into the Monolith NT.115 device (NanoTemper), and MST was measured using 40% (RED) light-emitting diode and medium MST power. Data were processed using the manufacturer’s software (MO.affinity 2.3, NanoTemper, https://nanotempertech.com/) following published protocols (*43*). Control experiments included measurements of the binding of the RED-tris-NTA dye to His-DJ-1 and of a dye-labeled AptamerCy5 to adenosine 5′-monophosphate using the manufacturer’s kit (Monolith NT.115 Control Kit RED, NanoTemper).

### Coimmunoprecipitation

Coimmunoprecipitation from human embryonic kidney cells was performed using Pierce Anti-HA Agarose (Thermo Fisher Scientific, 26181) following the manufacturer’s protocol. For in vitro pull-down, proteins were purified as described above. For recombinant protein pull-down, Ni-NTA–agarose (Thermo Fisher Scientific, R90101) was used following the manufacturer’s methods. Samples were then loaded onto a commercial SDS gel and subjected to electrophoresis and Western blotting.
