# Supplementary material for: Phosphoglycerate kinase is a central leverage point in Parkinson’s disease–driven neuronal metabolic deficits
Source: Sci Adv. 2024 Aug 21;10(34):eadn6016. doi: 10.1126/sciadv.adn6016 (PMC11338267; doi:10.1126/sciadv.adn6016)
Supplement: Supplementary file 1 — Figs. S1 to S5 [file sciadv.adn6016_sm.pdf]

Science Advances
AAAS

Supplementary Materials for

Phosphoglycerate kinase is a central leverage point in Parkinson's disease–driven neuronal metabolic deficits

Alexandros C. Kokotos *et al.*

Corresponding author: Timothy A. Ryan, taryan@med.cornell.edu

Sci. Adv. **10**, eadn6016 (2024)
DOI: 10.1126/sciadv.adn6016

This PDF file includes:

Figs. S1 to S5

Supplementary Materials

Data availability

Raw data for this study are openly available at 10.5281/zenodo.11122117.

Fig. S1 Synaptic PGK1 quantification.

(**A**) Ensemble average vGlutI-pH fluorescence in neurons expressing HK1 (black), AldoA (dark green), AldoC (light green), GAPDH (purple), PGK1 (red), PKM1 (brown) and 0.1 mM glucose control (blue). (**B**) The remaining fluorescence 55 s post stimulation after each train is plotted as mean ± SEM, 0.1 mM glucose control N=13, HK1 N=8, AldoA N=4, AldoC N=6, GAPDH N=4, PGK1 N=5, PKM1 N=7 (**C**) Immunostaining against PGK1 (red), a nerve terminal marker synI/II (magenta) and an axonal protein tubulin βIII (green). Scale bar 2 µm. Synaptic (synapsin I/II positive, white arrows) to axonal (synapsin I/II negative, blue arrows) fluorescence intensity ratio is quantified in (**D**) and shows significant enrichment of PGK1 in nerve terminals. mean ± SEM, syn I/II N=1370, Tubulin βIII N=1370, PGK1 N=600, GAPDH N-770, ***p < 0.001 1-way ANOVA. (**E**) Immunostaining against PGK1 in cultures from either female of male animals reveals no difference in the nerve terminal PGK1 content. mean ± SEM, Female N=500, Male N=500, ^{ns}p > 0.05 unpaired t-test (**F**) PGK1-HALO (labelled with JF585) also accumulates in nerve terminals (vGlutI-pH, visualized during NH_4Cl application) scale bar 6 µm. (**G**) Synaptic PGK1 was quantified in cells transfected with PGK1-HALO (red) compared to non-transfected cells (grey) at synapsin positive puncta. The construct leads to significant PGK1 overexpression as indicated by the cumulative histograms. PGK1-HALO N=93, non-transfected N=950 puncta, ****p* < 0.001 Kolmogorov-Smirnof. (**H**) Synaptic endurance score, measured as the fluorescence signal of recovery after the 10th round for each cell tested compared to the average synaptic PGK1-HALO expression normalized to non-transfected cells N=12. Dashed blue line shows the average synaptic endurance score for low glucose in control neurons after the 10th round. (**I**) Example of the PGK1-HALO transfected cells labeled with JF503 (green), immunostained against PGK1 (red) and synapsin I/II (magenta). Scale bar 6 µm. (**J**) vGlutI-pH trace of the lowest PGK1-HALO expressing amount (light red, PGK1 2-fold overexpression, noted as lighter red large point Fig. S1H) compared to average control (blue).

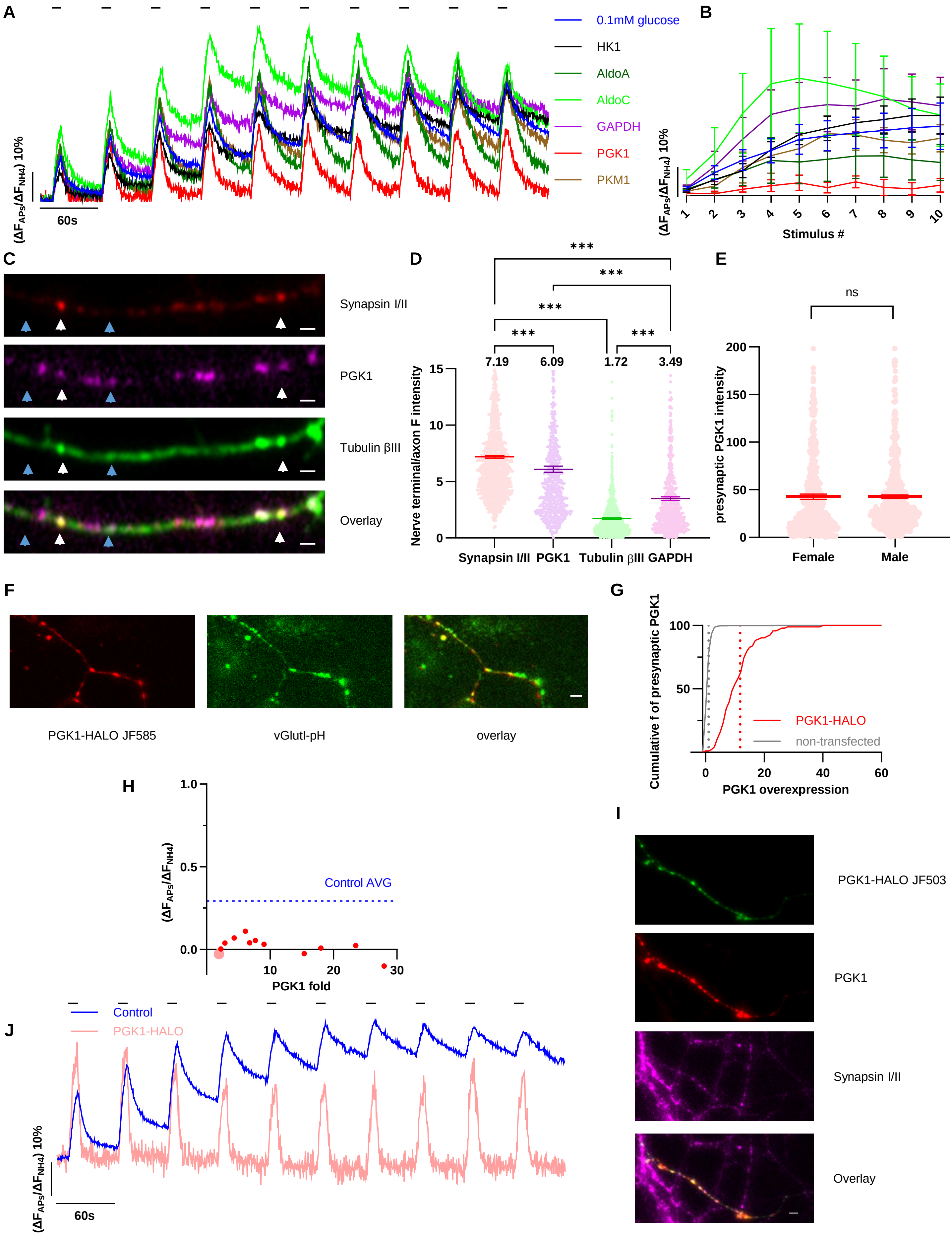

A
0.1mM glucose
HK1
AldoA
AldoC
GAPDH
PGK1
PKM1
60s
B
Stimulus #
C
Synapsin I/II
PGK1
Tubulin βIII
Overlay
D
Nerve terminal/axon F intensity
7.19
6.09
1.72
3.49
Synapsin I/II
PGK1
Tubulin βIII
GAPDH
E
ns
presynaptic PGK1 intensity
Female
Male
F
PGK1-HALO JF585
vGlutl-pH
overlay
G
Cumulative f of presynaptic PGK1
PGK1-HALO
non-transfected
PGK1 overexpression
H
Control AVG
PGK1 fold
I
PGK1-HALO JF503
PGK1
Synapsin I/II
Overlay
J
Control
PGK1-HALO
60s

Figure S1

Fig. S2 Terazosin driven metabolic resilience requires PGK1.
(**A**) Quantification of KD efficiency of PGK1 from transfected cells with a PGK1 shRNA (yellow) and non-transfected (grey) and stained against PGK1 shows that PGK1 was reduced by 70% compared to controls. Mean ± SEM, non-transfected N=37, PGK1 shRNA N=10, $^{**}p < 0.01$ unpaired t-test. (**B**) Representative images of a cell expressing PGK1 shRNA and mTagBFP2 (cyan, white arrow) and stained against PGK1 (magenta). Non-transfected cells are noted by blue arrows. scale bar 6 µm (**C**) Ensemble average vGlutI-pH traces in 5 mM glucose control (teal) compared to PGK1 KD (yellow) stimulated with 600 APs (10 Hz, indicated by the black bar). (**D**) Quantification of the remaining fluorescence of the vGlut-pH traces 60 s post stimulation (indicated by the dotted line in **C**) mean ± SEM, control N=7, PGK1 KD N=8, $^{*}p < 0.05$ unpaired t-test. (**E**) KD of PGK1 (yellow) abolishes the Terazosin (TZ) hypometabolic rescue (green) in 0.1 mM glucose. (**F**) Quantification of the remaining fluorescence from (**E**). mean ± SEM, 10 µM TZ N=10, 10 µM TZ + PGK1 KD N=14, $^{*}p < 0.05$, $^{**}p < 0.01$ 2-way ANOVA. (**G**) Terazosin-md that binds PGK1 but not a1R (purple) still offers protection in 0.1 mM glucose. (**H**) Quantification of the remaining fluorescence from (**G**). mean ± SEM, 10 µM TZ N=10, 10 µM TZ-md N=10, $^{ns}p > 0.05$ 2-way ANOVA.

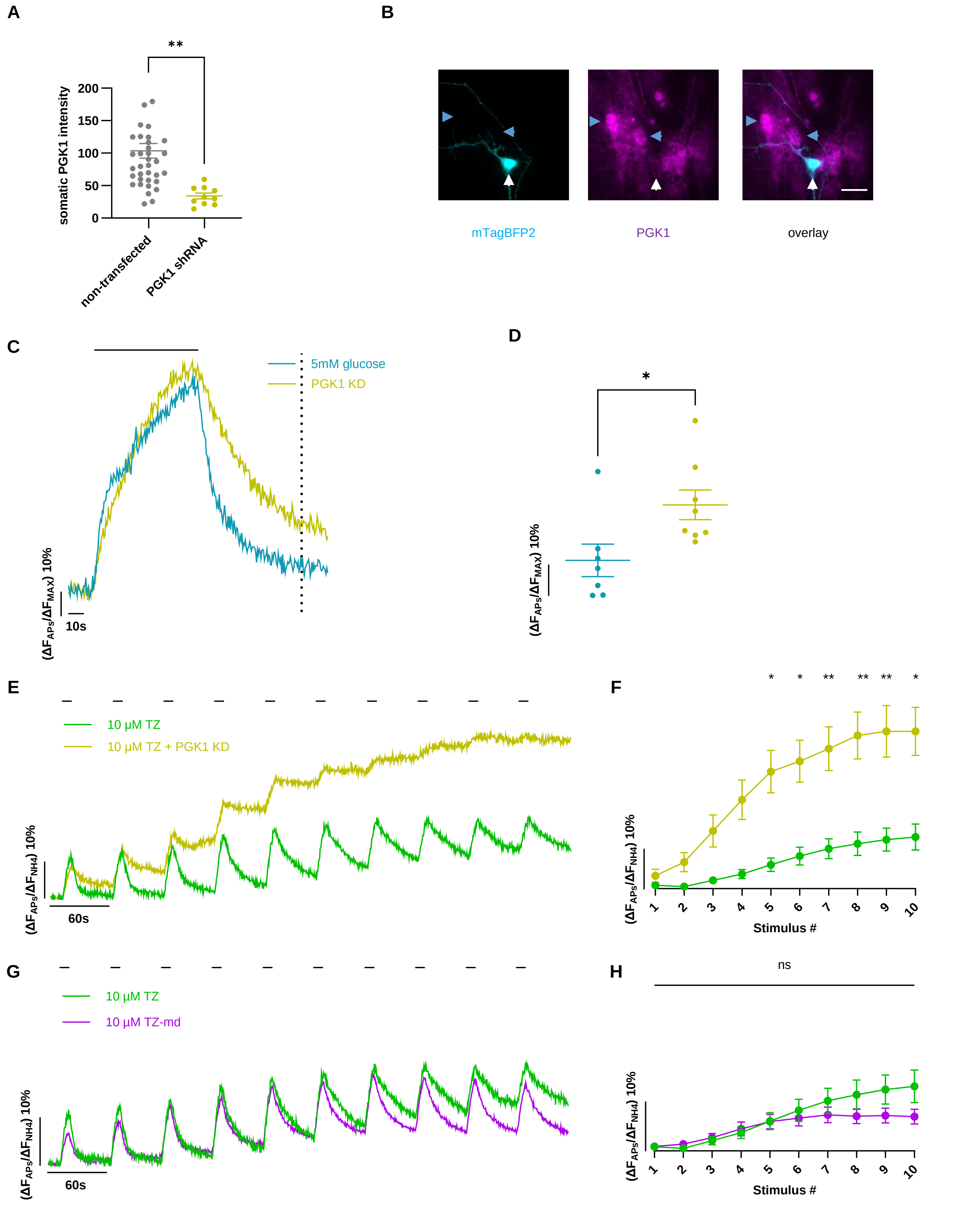
A
**
somatic PGK1 intensity
200
150
100
50
0
non-transfected
PGK1 shRNA
B
mTagBFP2
PGK1
overlay
C
5mM glucose
PGK1 KD
(ΔFAPs/ΔFMAX) 10%
10s
D
*
(ΔFAPs/ΔFMAX) 10%
E
10 µM TZ
10 µM TZ + PGK1 KD
(ΔFAPs/ΔFNH4) 10%
60s
F
* * ** ** ** *
(ΔFAPs/ΔFNH4) 10%
1 2 3 4 5 6 7 8 9 10
Stimulus #
G
10 µM TZ
10 µM TZ-md
(ΔFAPs/ΔFNH4) 10%
60s
H
ns
(ΔFAPs/ΔFNH4) 10%
1 2 3 4 5 6 7 8 9 10
Stimulus #

Figure S2

Fig. S3 PGK1 can rescue ATP dynamics even in absence of mitochondrial ATP.

(**A**) Ensemble average synapto-iATPSnFR2-miRFP670nano3 traces for ATP sensor (blue) and synapto-cpsfGFP-miRFP670nano3 non-ATP binding sensor (grey) transfected cells stimulated with 600 APs at 10 Hz in 0.1 mM glucose. The non-ATP binding control sensor is unresponsive to electrical stimulation. (**B**) Comparison of sensor responses normalized values at the end of the stimulus and (**C**) 50 s post-stimulus, mean ± SEM indicated, synapto-iATPSnFR2-miRFP670nano3 N=12, synapto-cpsfGFP-miRFP670nano3 N=9, ***$p < 0.001$ one-way ANOVA. (**D**) Ensemble average synapto-iATPSnFR2-miRFP670nano3 traces for 5 mM glucose control in presence of 2 μM Oligomycin (teal) and PGK1-HALO (red) transfected cells stimulated with 600 APs at 10 Hz. PGK1-HALO neurons show a significant activity dependent upregulation of ATP synthesis following activity. Comparison of (**E**) and normalized values at the end of the stimulus and (**F**) 50 s post-stimulus. mean ± SEM indicated, 5 mM glucose Oligomycin control N=11, PGK1-HALO N=10, **$p < 0.01$ unpaired t-test. (**G**) Quantification of the DJ-1 KD efficiency shows DJ-1 is reduced by 30%. mean ± SEM, Control N=11, DJ-1 shRNA N=8, *$p < 0.05$ unpaired t-test. (**H**) Representative images of a cell expressing PGK1 shRNA and mTagBFP2 (cyan, white arrow) and stained against DJ-1 (magenta). Non-transfected cells are noted by blue arrows. scale bar 6 μm(**I**) The DJ-1 KD (black) slowing of SV endocytosis in 0.1 mM glucose can be rescued be reintroduction of shRNA resistant DJ-1 (orange), but not when C106 is mutated (magenta). (**J**) Quantification of the remaining fluorescence 60 s post stimulation (highlighted by dotted line in (**I**) mean ± SEM, 0.1 mM glucose control N=16, DJ-1 KD N=12, DJ-1 KD + DJ-1 N=9, DJ-1 KD + DJ-1 C106A N=11, $^{ns}p > 0.05$, *$p < 0.05$ one-way ANOVA.

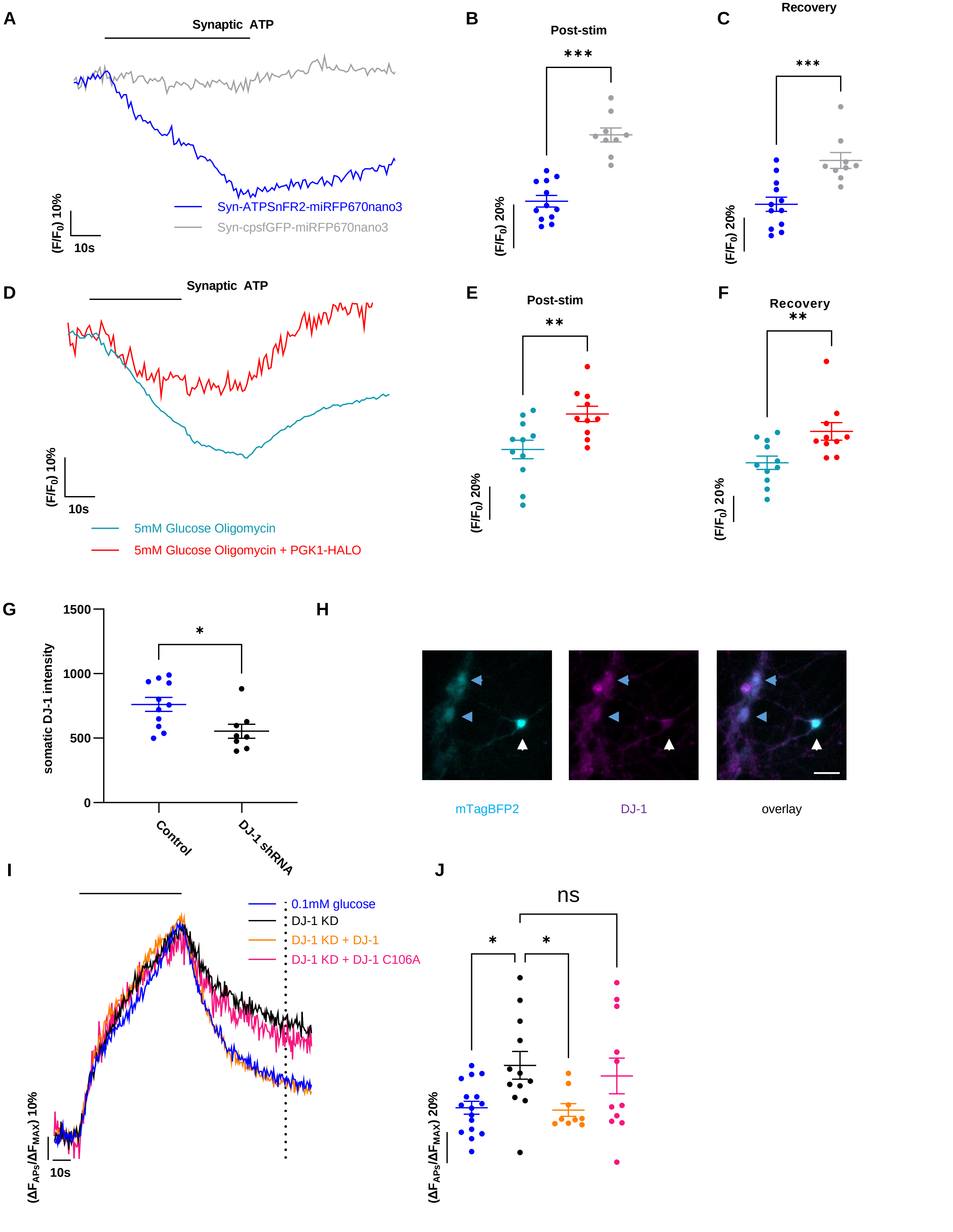
A
Synaptic ATP
(F/F0) 10%
10s
Syn-ATPSnFR2-miRFP670nano3
Syn-cpsfGFP-miRFP670nano3
B
Post-stim
***
(F/F0) 20%
C
Recovery
***
(F/F0) 20%
D
Synaptic ATP
(F/F0) 10%
10s
5mM Glucose Oligomycin
5mM Glucose Oligomycin + PGK1-HALO
E
Post-stim
**
(F/F0) 20%
F
Recovery
**
(F/F0) 20%
G
somatic DJ-1 intensity
1500
1000
500
0
*
Control
DJ-1 shRNA
H
mTagBFP2
DJ-1
overlay
I
0.1mM glucose
DJ-1 KD
DJ-1 KD + DJ-1
DJ-1 KD + DJ-1 C106A
(ΔFAPs/ΔFMAX) 10%
10s
J
ns
*
*
(ΔFAPs/ΔFMAX) 20%

Figure S3

Fig. S4 DJ-1 is required for PGK1 mediated synaptic resilience.
(**A**) Ensemble vGlutI-pH traces in neurons, expressing PGK1-HALO (red) or PGK1-HALO with DJ-1 KD (dark red) and (**C**) TZ treated neurons (green) versus TZ treated in DJ-1 KD (dark green) subjected to repeated electrical stimulation in 0.1 mM glucose. (**B**) Synaptic endurance measured vGlutI-pH fluorescence 55 s after each stimulus bout for the traces in (**A**) mean ± SEM, PGK1-HALO N=12, PGK1-HALO + DJ-1 KD N=7, **p < 0.01, ***p < 0.001 2-way ANOVA and (**D**) mean ± SEM, TZ N=10, TZ + DJ-1 KD N=10, **p* < 0.05, ****p* < 0.001 mixed-effects (**E**) co-IP of PGK1-HA and myc-DJ-1 from HEK293T cells against HA shows that PGK1 can pull-down DJ-1. The pull down can be detected when either PGK1-HA and myc-DJ-1 were co-transfected in the HEK cells (PGK1-DJ-1) or when the lysates from single transfected cells were mixed *in-vitro* (PGK1 + DJ-1). (**F**) co-IP of purified recombinant His-DJ-1 and untagged PGK1 *in-vitro* shows that DJ-1 can pull-down PGK1. As an independent control, His-GAPDH was used, which can also pull-down PGK1. Red asterisks denote non-specific bands of used antibodies. (**G**) Ensemble average vGlutI-pH traces in response to a single 100 AP burst (10 Hz, indicate by black bar) are similar in 5 mM glucose control (blue) and DJ-1 KD neurons (black), but (**H**) are significantly slowed with a combination of 0.1 mM glucose and 2 µM Oligomycin. (**I**) Ensemble average vGlutI-pH traces in response to a single 100 AP burst are similar in 1.25 mM Lactate and Pyruvate control and DJ-1 KD neurons. (**J**) Fluorescence recovery 10 s post stimulus for the traces in (**G, H, I**) mean ± SEM, High glucose Control N=6, High glucose DJ-1 KD N=11, Low glucose Oligomycin Control N=6, Low glucose Oligomycin + DJ-1 KD N=5, Lactate Pyruvate Control N=7, Lactate Pyruvate + DJ-1 KD N=8, $p^{ns} > 0.05$, *p < 0.05 unpaired t-test.

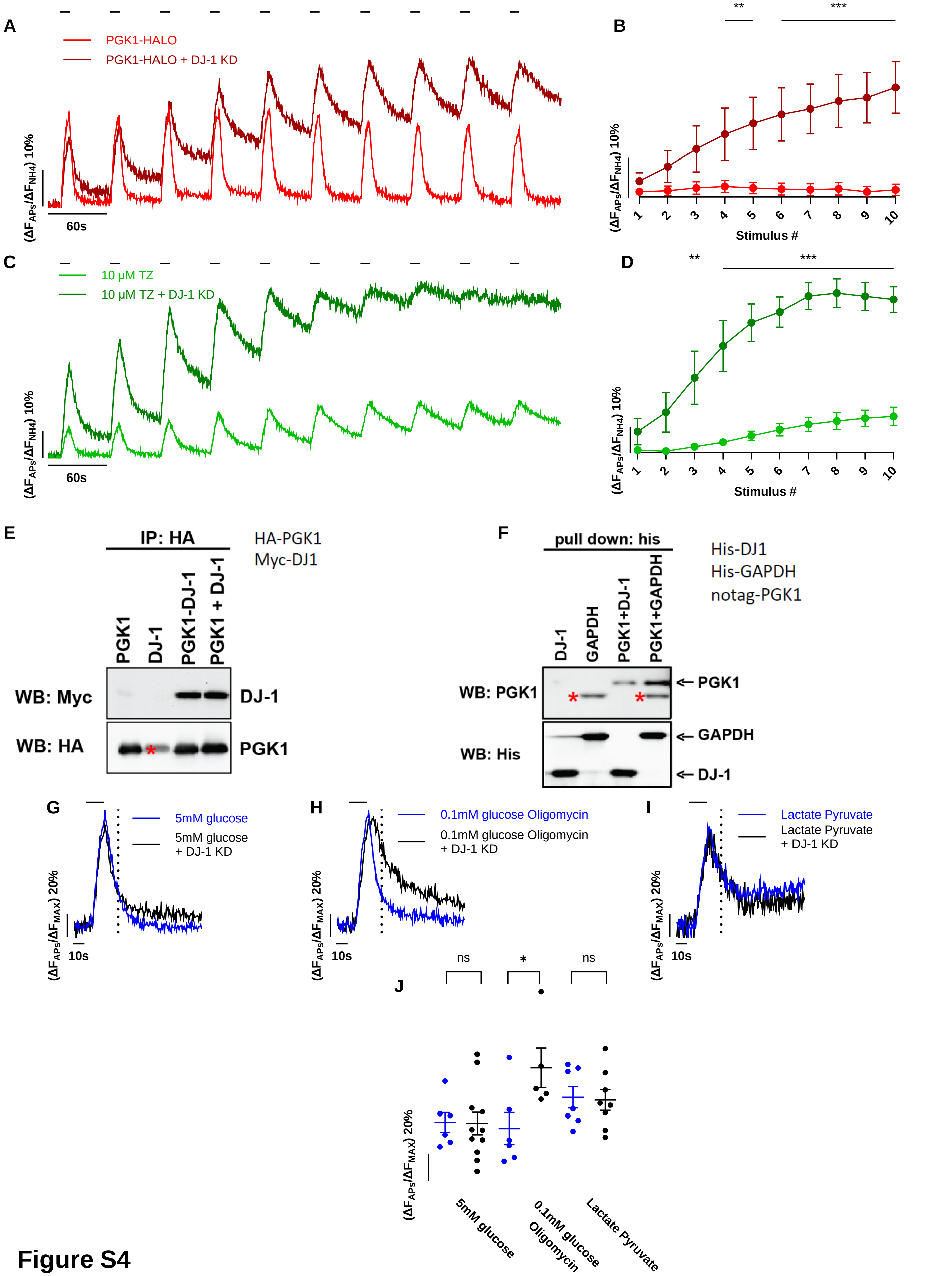
A
PGK1-HALO
PGK1-HALO + DJ-1 KD
(ΔFAPs/ΔFNH4) 10%
60s
B
**
***
(ΔFAPs/ΔFNH4) 10%
Stimulus #
C
10 μM TZ
10 μM TZ + DJ-1 KD
(ΔFAPs/ΔFNH4) 10%
60s
D
**
***
(ΔFAPs/ΔFNH4) 10%
Stimulus #
E
IP: HA
HA-PGK1
Myc-DJ1
PGK1
DJ-1
PGK1-DJ-1
PGK1 + DJ-1
WB: Myc
DJ-1
WB: HA
PGK1
F
pull down: his
His-DJ1
His-GAPDH
notag-PGK1
DJ-1
GAPDH
PGK1+DJ-1
PGK1+GAPDH
WB: PGK1
PGK1
GAPDH
WB: His
DJ-1
G
5mM glucose
5mM glucose + DJ-1 KD
(ΔFAPs/ΔFMAX) 20%
10s
H
0.1mM glucose Oligomycin
0.1mM glucose Oligomycin + DJ-1 KD
(ΔFAPs/ΔFMAX) 20%
10s
I
Lactate Pyruvate
Lactate Pyruvate + DJ-1 KD
(ΔFAPs/ΔFMAX) 20%
10s
J
ns
*
ns
(ΔFAPs/ΔFMAX) 20%
5mM glucose
0.1mM glucose Oligomycin
Lactate Pyruvate

Figure S4

Fig. S5 PGK1 *in-vivo* expression protects dopaminergic neurons from degeneration.
(**A**) Immunostaining of control and AAV PGK1 mid-brain brain slices against a nuclear marker, DAPI, mRuby and a dopaminergic neuronal marker, DAT. Scale bar 100 µm.

A

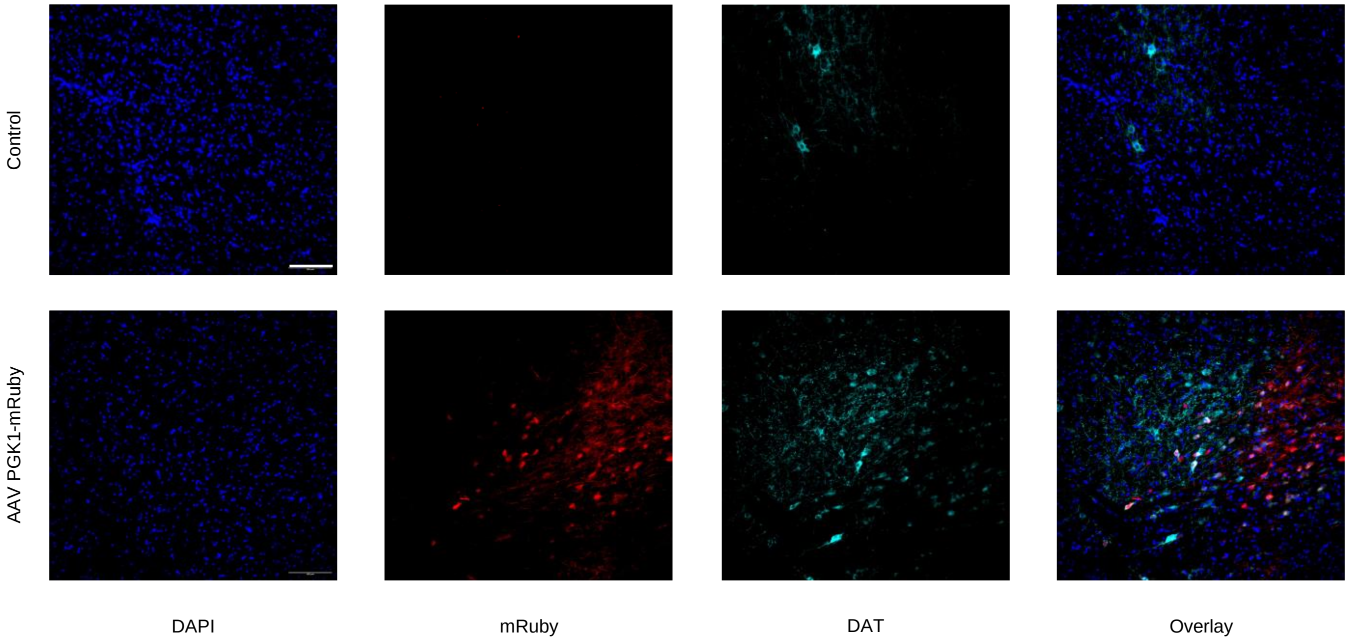

Figure S5
